# The impact of epigenetics on tumor metabolism: Friend or foe in drug response?

**DOI:** 10.3389/freae.2026.1755829

**Published:** 2026-02-09

**Authors:** Serena Castelli, Melania Franchini, Luigi Ferraro, Nicolò Mangraviti

**Affiliations:** 1 Department for the Promotion of Human Science and Quality of Life, San Raffaele Open University, Rome, Italy; 2 Department of System Biology, Irving Cancer Research Center, Columbia University, New York, NY, United States; 3 Sylvester Comprehensive Cancer Center, University of Miami Miller School of Medicine, Miami, FL, United States; 4 Department of Public Health Sciences, University of Miami Miller School of Medicine, Miami, FL, United States; 5 Department of Neuroscience and Physiology, NYU Grossman School of Medicine, NYU Langone Health, New York, NY, United States

**Keywords:** drug repurposing, drug resistance, metabolic reprogramming, non-coding RNA, post translation modifications

## Abstract

Cancer cells exhibit remarkable plasticity, enabling them to survive therapeutic pressure by dynamically rewiring both their epigenetic landscape and metabolic circuitry. Emerging evidence reveals that epigenetic mechanisms, including DNA methylation, histone modifications, chromatin remodeling, and non-coding RNAs, are tightly coupled to metabolic pathways through key metabolites that function as cofactors or regulators of chromatin-modifying enzymes. This reciprocal interplay establishes self-reinforcing loops that sustain tumor growth, promote heterogeneity, and drive the emergence of drug-tolerant states. In this review, we summarize current knowledge on how epigenetic remodeling shapes metabolic reprogramming and, in turn, how altered metabolite pools influence chromatin states in cancer. We highlight the central role of long non-coding RNAs and other ncRNA species in coordinating epigenetic and metabolic adaptations that underpin therapy resistance. We further examine the contribution of metabolite-dependent post-translational modifications, such as acetylation, methylation, lactylation, and succinylation, to the regulation of tumor aggressiveness and treatment response. Finally, we discuss how multi-omics integration, computational network approaches, and AI-enabled modeling are accelerating the discovery of epigenetic–metabolic vulnerabilities and informing the development of precision therapeutic strategies. Understanding and targeting this epigenetic–metabolic axis holds substantial promise for overcoming drug resistance and improving the durability of cancer therapies.

## 1 Introduction

Cancer remains one of the leading causes of mortality worldwide despite major advances in detection and therapy (Bray et al., 2024). A key reason for its persistence is the exceptional plasticity of malignant cells, which remodel transcriptional programs and metabolic fluxes to sustain growth, evade stress, and acquire drug resistance. Central to this adaptability is the reciprocal interaction between epigenetic regulation and cellular metabolism, a dynamic interface that integrates environmental cues into gene expression and biochemical output to shape tumor behavior (Mangraviti and Castelli, 2025). In this context, epigenetic mechanisms such as DNA methylation, histone modifications, chromatin remodeling, and the activity of non-coding RNAs (ncRNAs) do not merely annotate the genome; they function as sensors and effectors that couple nutrient status, redox balance, and bioenergetic demand to transcriptional states that promote survival under therapeutic pressure.

Metabolic rewiring is also a defining hallmark of cancer and involves enhanced aerobic glycolysis, reprogrammed tricarboxylic acid (TCA) cycle activity, and extensive remodeling of lipid and amino acid pathways (Pang and Wu, 2025). These alterations not only sustain biomass synthesis but also generate metabolites, including acetyl-CoA, S-adenosylmethionine (SAM), NAD^+^, and α-ketoglutarate that serve as cofactors or inhibitors of chromatin-modifying enzymes (Zhang and Jagannath, 2025). Consequently, metabolism directly shapes chromatin state and transcription, while epigenetic remodeling reciprocally regulates metabolic gene expression. This bidirectional feedback establishes self-reinforcing loops that promote tumor heterogeneity, disease progression, and resistance to therapy.

Within this regulatory network, non-coding RNAs, including microRNAs (miRNAs), long non-coding RNAs (lncRNAs), and circular RNAs (circRNAs), have emerged as pivotal mediators that connect chromatin-modifying complexes such as PRC2, HDACs, and DNMTs with metabolic pathways. Acting as guides, scaffolds, or decoys, ncRNAs fine-tune metabolic flux, redox homeostasis, and stress responses, thereby influencing therapeutic sensitivity and resistance (Mangraviti and De Windt, 2022). Through these interactions, ncRNAs form a central component of the epigenetic–metabolic circuitry that defines tumor adaptability and clinical outcome.

Clinically, the epigenetic–metabolic axis offers promising therapeutic opportunities. Inhibitors of DNA methyltransferases and histone deacetylases, as well as drugs targeting mutant metabolic enzymes such as IDH1 and IDH2, demonstrate that simultaneous modulation of chromatin and metabolism can alter tumor fate (Issa and DiNardo, 2021). More broadly, strategies aimed at disrupting feedback between metabolic and epigenetic regulators hold great potential for overcoming resistance and improving treatment efficacy.

In this review, we summarize the fundamental mechanisms of epigenetic regulation in cancer, outline the major features of metabolic reprogramming, and discuss how their convergence drives adaptive plasticity while revealing exploitable vulnerabilities. We also highlight bioinformatic and multi-omics approaches that are redefining the discovery of epigenetic–metabolic targets and guiding the development of next-generation combination therapies in precision oncology.

## 2 Molecular mechanisms and epigenetic regulation via non-coding RNAs

Within the intricate interplay between epigenetic regulation and metabolic remodeling in therapeutic response, ncRNAs have emerged as key modulators capable of influencing gene expression programs and shifting drug sensitivity toward either cell death or survival. These small regulatory RNAs can target multiple downstream effectors and dynamically respond to therapeutic pressure, thereby modulating cellular adaptive mechanisms. Another particularly compelling aspect is the enrichment of ncRNAs within exosomes, which positions them as key mediators of communication within the tumor microenvironment. Exosomes and other extracellular vesicles serve as critical vehicles for intercellular information transfer, facilitating the exchange of regulatory molecules between cancer cells and the diverse stromal and immune cell populations in their vicinity. Through this vesicle-mediated crosstalk, ncRNAs can profoundly influence tumor progression, metabolic and epigenetic adaptation, and ultimately the response to therapy (Jin and Bai, 2025; Chen et al., 2021). Over the past few years, research on the contribution of ncRNAs to therapy response has expanded substantially to evaluate their potential as targets for combination treatment strategies.

### 2.1 Long non-coding RNAs and therapeutic response in the epigenetic–metabolic axis

Long non-coding RNAs (lncRNAs, classically defined as transcripts >200 nt, although some sources apply a >500 nt cutoff) have emerged as essential regulators of tumor plasticity by bridging chromatin organization, metabolic homeostasis, and therapeutic outcome (Huarte, 2015). Acting as molecular scaffolds, decoys, and regulatory hubs, lncRNAs fine-tune transcriptional and metabolic networks that allow cancer cells to adapt to environmental fluctuations and pharmacological stress. Depending on the cellular context and tumor type, lncRNAs can either reinforce therapy resistance or restore sensitivity, placing them at the center of the epigenetic–metabolic circuitry that determines treatment efficacy (Grossi et al., 2025; Wu et al., 2023). A growing body of evidence demonstrates that oncogenic lncRNAs coordinate epigenetic remodeling with metabolic reprogramming to maintain proliferation, redox balance, and survival during therapy (Figure 1) (Grossi et al., 2025).

**FIGURE 1:**
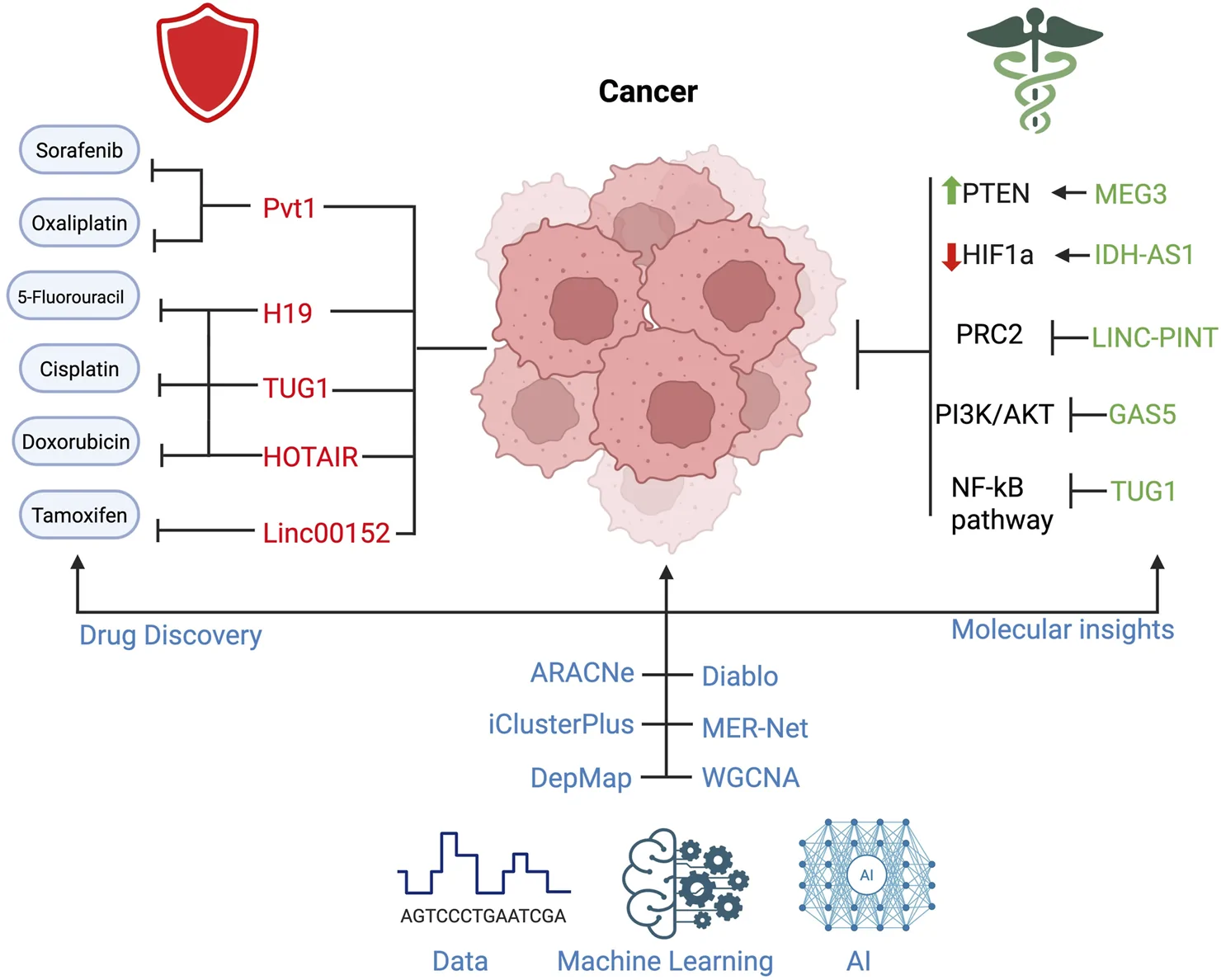
Summary figure of the relationship between lncRNAs and drug resistance and discovery. Bioinformatic tools can be key for identifying this relationship by comparing the gene expression profiles of resistant and non-resistant cells.

At the mechanistic level, many of the earliest and most pervasive effects of lncRNAs in cancer emerge through their ability to shape chromatin architecture and epigenetic states. By guiding chromatin-modifying complexes, organizing nuclear domains, and establishing repressive or permissive transcriptional environments (Jiang et al., 2024), lncRNAs lay the foundation upon which downstream metabolic and signaling adaptations are built. This positioning at the interface of chromatin organization and transcriptional control enables lncRNAs to act as primary regulators of cellular plasticity, making epigenetic scaffolding functions a logical starting point for understanding their role in therapy response.

#### 2.1.1 LncRNAs as integrators of chromatin scaffolds and epigenetic regulators

Several lncRNAs exert their primary effects by directly shaping chromatin structure and recruiting epigenetic modifiers, thereby establishing transcriptional programs that favor tumor adaptation. HOTAIR exemplifies this function by recruiting PRC2 and LSD1 to specific promoters (Bhan et al., 2013), enforcing repressive histone marks such as H3K27me3 and H3K4me2 demethylation on tumor suppressors including PTEN (Li et al., 2013). Through this coordinated chromatin repression, HOTAIR establishes a transcriptional environment that favors metabolic rewiring. This mechanism mirrors the activity of DANCR against FBP1. These epigenetic events suppress oxidative phosphorylation while enhancing glycolysis, thereby linking chromatin remodeling directly to altered bioenergetic states, elevating NADPH and glutathione levels that sustain resistance to cisplatin and doxorubicin in breast (Uslu et al., 2025), ovarian (Wu et al., 2022), and lung cancers (Ashton et al., 2018).

NEAT1 also acts at the chromatin level by recruiting EZH2 to deposit H3K27me3 and repress lineage-specific genes. In colorectal cancer, NEAT1 upregulates SIRT1 including via miR-34a sponging, while in hepatocellular carcinoma it regulates mTOR-dependent paraspeckle-mediated splicing (Pisani and Baron, 2020; Park et al., 2021). These chromatin and post-transcriptional activities converge on pathways controlling cellular metabolism. The SIRT1–PGC-1α axis downstream of NEAT1 is a canonical driver of mitochondrial biogenesis (Mihaylov et al., 2023).

LINC-PINT, a p53-induced nuclear lncRNA, interacts with PRC2 and its conserved PINT87aa micropeptide to repress oncogenic transcriptional programs and invasion (Marín-Béjar et al., 2013; Marín-Béjar et al., 2017; Lin et al., 2024). By restricting oncogenic transcription, LINC-PINT indirectly constrains metabolic and proliferative programs.

FENDRR is frequently downregulated in gastric and other cancers, where its restoration inhibits migration, invasion, EMT, and inflammation-linked metabolic stress through modulation of PRC2-dependent chromatin programs and attenuation of TGF-β and NF-κB signaling (Li F. et al., 2023). Here again, epigenetic repression is functionally coupled with reduced inflammatory and metabolic pressure.

#### 2.1.2 lncRNAs in metabolic enzyme regulation and pathway control

Beyond chromatin scaffolding, many lncRNAs directly engage metabolic enzymes and signaling pathways, enabling tight coordination between transcriptional control and bioenergetic output (Table 1). MALAT1 functions as a molecular bridge between chromatin remodeling and metabolic and translational control. It forms a ternary HDAC9–MALAT1–BRG1 complex that promotes H3K27me3-associated repression of contractile genes (Lino et al., 2018) and recruits BRG1 to inflammatory cytokine promoters such as IL6 and CXCL8 in hepatocellular carcinoma. Through these chromatin-level interactions, MALAT1 primes cells for downstream metabolic adaptation, including the stabilization of nuclear SREBP-1c to enhance lipogenesis (Yan et al., 2016). This lipogenic shift is further reinforced by its effects on mitochondrial quality control. In cancer cells, MALAT1 elevates and modulates PINK1-dependent mitophagy to rewire oxidative TCF7L2 through SRSF1 and mTORC1–4EBP1 activation (Zhao et al., 2021), inducing glycolytic enzymes and repressing gluconeogenesis (Malakar et al., 2019), thereby coordinating mitochondrial turnover with glycolytic flux, and regulates metabolic programs in prostate cancer through a MYBL2/mTOR pathway (Mu et al., 2022). Its broader role in tumor progression and therapy resistance is supported by metabolic signaling through mTOR and PKM2 (Bitaraf et al., 2025), integrating transcriptional, translational, and metabolic control, and evidence from multiple myeloma showing that antisense-mediated MALAT1 inhibition downregulates CD38 and alters energy metabolism via PRC2.

**TABLE 1:** Summary table of the mechanisms through which lncRNAs can regulate drug resistance.

Drug/Therapy	lncRNA(s)	Final mechanism	Key references
Cisplatin/Doxorubicin	HOTAIR	Increased glycolysis and antioxidant capacity	Bhan et al. (2013), Li et al. (2013), Uslu et al. (2025)
Cisplatin	NEAT1	Enhanced homologous recombination repair	Pisani and Baron (2020), Zhu et al. (2020)
Cisplatin/5-FU	H19	Glycolytic activation supporting survival	Chen et al. (2017)
Cisplatin	TUG1	Increased ROS	He et al. (2024)
5-Fluorouracil (5-FU)	H19	Elevated glycolysis	Wang et al. (2018)
EGFR-TKIs	LINC00963	Activation of PI3K–AKT–mTOR signaling	Saberiyan et al. (2025), Xie et al. (2022)
TKIs	H19	Metabolic adaptation enabling survival	Wang et al. (2018)
PARP inhibitors	NEAT1	Increased homologous recombination → PARP-i resistance	Liu and Liu (2024)
Anti-angiogenic therapy	PVT1	MYC/STAT3 activation	Onagoruwa et al. (2020)
Sorafenib	PVT1	Epigenetic and metabolic remodeling	Onagoruwa et al. (2020)
Oxaliplatin	PVT1	Enhanced glycolysis and glutamine metabolism	Onagoruwa et al. (2020), Cacace et al. (2017)
Tamoxifen	LINC00152	Suppressed ferroptosis	Saatci et al. (2024)
Azacitidine/DNMT1-i	MEG3	Reactivation of p53/PTEN tumor suppressor pathway	Li et al. (2016), Zhu et al. (2015), Lin et al. (2021)
PI3K/mTOR inhibitors	GAS5	Increased sensitivity via mTOR-autophagy regulation	Patel et al. (2023), Sang et al. (2021)

PVT1 stabilizes MYC and STAT3, enhancing transcription of glycolytic enzymes such as HK2 and LDHA and promoting glutamine metabolism (Johnsson and Morris, 2014; Xu et al., 2019). Through MYC and STAT3 stabilization, PVT1 directly couples transcriptional amplification to metabolic demand. PVT1 amplification correlates with resistance to oxaliplatin, sorafenib, and anti-angiogenic therapies (Onagoruwa et al., 2020), reflecting its capacity to assemble EZH2- and DNMT1-dependent chromatin complexes that silence pro-apoptotic genes and maintain SAM pools (Nylund et al., 2024). Thus, metabolic activation and epigenetic repression are functionally aligned.

H19, an imprinted lncRNA induced by hypoxia and high glucose (Xu et al., 2021), upregulates PDK1 and increases expression of glycolytic enzymes including PGK1 and HK2 (Chen et al., 2023). In this context, environmental cues are directly translated into metabolic reprogramming. This glycolytic switch contributes to resistance to 5-fluorouracil and tyrosine-kinase inhibitors in gastric and hepatocellular carcinomas, and high H19 expression correlates with poor survival (Chen et al., 2017). Here, metabolic adaptation and therapeutic escape are mechanistically linked.

LINC00963 stabilizes PGK1, blocks its ubiquitination, activates PI3K–AKT–mTOR signaling, enhances aerobic glycolysis, and is associated with EGFR-TKI resistance in NSCLC (Saberiyan et al., 2025; Xie et al., 2022). By protecting a key glycolytic enzyme, LINC00963 sustains pathway activation.

GAS5 regulates PI3K/AKT/mTOR signaling, influences TCA-cycle flux as a mitochondria-associated lncRNA, and fine-tunes mTOR-dependent autophagy, enhancing apoptosis and sensitivity to PI3K/mTOR inhibitors and chemotherapeutics (Patel et al., 2023; Sharma et al., 2019; Sang et al., 2021). In contrast, GAS5 couples metabolic restraint to increased therapeutic vulnerability.

By contrast, several lncRNAs function as tumor-suppressive or metabolic checkpoint molecules that restore sensitivity. MEG3 is frequently silenced by DNMT1-mediated hypermethylation, and DNMT1 inhibition or azacitidine treatment restores its expression and reactivates p53-dependent tumor-suppressive pathways including PTEN, contributing to apoptosis and to the anti-tumor effects of hypomethylating agents (Li et al., 2016).

IDH1-AS1 enhances IDH1 activity, increases α-ketoglutarate and reduces HIF-1α stabilization, thereby shifting metabolism away from glycolysis and restraining glioma growth (Xiang et al., 2018). This metabolic shift is mechanistically linked to reduced hypoxic signaling, the overexpression of this lncRNA inhibits glioma proliferation, induces apoptosis, arrests the cell cycle and suppresses tumorigenesis *in vivo* (Wang J. et al., 2020).

#### 2.1.3 lncRNAs as miRNA sponges and post-transcriptional regulators

In addition to epigenetic scaffolding and metabolic pathway control, lncRNAs exert a major layer of regulation at the post-transcriptional level. By acting as miRNA sponges and organizing RNA–protein complexes, they fine-tune gene expression programs that directly impact stress responses and therapy sensitivity.

NEAT1 forms paraspeckles that sequester tumor-suppressive miRNAs and transcriptional repressors (Pisani and Baron, 2020). Through this nuclear compartmentalization, NEAT1 reshapes post-transcriptional control resulting in mediating cisplatin resistance in ovarian cancer via miR-770-5p/PARP1 (Zhu et al., 2020) and promoting homologous-recombination repair and PARP-inhibitor resistance through RAD51 and FOXM1 (Liu and Liu, 2024). Thus, miRNA sequestration is functionally associated with DNA repair capacity.

LINC00152 promotes therapy resistance by forming a KLF5-dependent transcriptional loop in breast cancer (Li et al., 2021), by regulating miR-139-5p/NOTCH1 in colorectal cancer, and by supporting EZH2-dependent proliferation and invasion in mesothelioma (Bian et al., 2017). Regarding the therapy response, it suppresses tamoxifen-induced ferroptosis via a cAMP/Ca^2+^ axis in ER + breast cancer (Saatci et al., 2024).

TUG1 enhances glycolysis and tumor progression. The presence of TUG1 in CAF-derived exosomal it has been demonstrated to be able to promote glycolysis via the miR-524-5p/SIX1 axis in hepatocellular carcinoma (Lu et al., 2022). This illustrates how stromal communication feeds into metabolic regulation. Reduced TUG1 expression in NSCLC impairs ROS clearance and increases vulnerability to platinum-based therapy (Guo et al., 2019).

Collectively, these findings position lncRNAs among the major determinants of therapeutic resilience. They influence drug uptake, oxidative and metabolic stress buffering, DNA repair capacity, apoptotic signaling, and tumor–immune interactions. Across these mechanisms, lncRNAs repeatedly emerge as integrators rather than isolated effectors. Clinical studies increasingly show that lncRNA signatures outperform coding gene panels in predicting responses to platinum agents, kinase inhibitors, PARP inhibitors, immunotherapy, and epigenetic drugs (Grillone et al., 2024; Chen et al., 2022; Fathi, 2020). Despite this progress, major challenges persist, including strong tissue specificity, nuclear localization, large chromatin-bound interactomes, and limited clinical assay standardization.

Advances in antisense oligonucleotides, siRNAs, CRISPR interference, and plasmid-based lncRNA therapies such as H19-DTA, together with improved nanoparticle and viral delivery strategies and high-resolution single-cell and spatial profiling, are now enabling precise targeting of lncRNA vulnerabilities. These technological advances directly address the mechanistic complexity outlined above. As mechanistic understanding continues to expand, lncRNAs represent a powerful but underexploited class of therapeutic targets with significant potential to overcome drug-resistant disease.

## 3 Tumor metabolism–functional consequences of epigenetic remodeling

The remodeling of the epigenetic landscape and metabolic reprogramming are both established hallmarks of cancer (Xu et al., 2023). While metabolic reprogramming has been recognized for decades—primarily due to its association with the altered bioenergetic and biosynthetic demands of cancer cells compared to their normal counterparts (Van et al., 2009)—the role of epigenetic alterations in tumorigenesis has emerged more recently as a critical area of investigation (Yu et al., 2024). A growing body of evidence highlights the tight interplay between cancer cell molecular biology, particularly epigenetic regulation and consequent gene expression modulation, and cellular biochemistry. This connection is largely mediated by metabolites that, beyond their conventional metabolic functions, act as key cofactors or substrates for epigenetic enzymes, thereby directly linking metabolic state to chromatin dynamics and transcriptional control (Wu et al., 2024). As a result, the metabolic alterations occurring in cancer cells not only reshape cellular metabolism but also influence the epigenetic landscape by modulating the availability of these key metabolites. This intricate metabolic–epigenetic crosstalk underscores the potential of targeting specific metabolites or metabolic pathways as a novel and promising strategy in cancer therapy.

The epigenetic consequences of metabolite modulation can affect DNA, RNA, and supercoiled DNA within chromatin. Among the most extensively studied and widely distributed epigenetic modifications are methylation and acetylation, which occur on DNA, RNA, and histone proteins within chromatin. These modifications are metabolite-dependent, relying primarily on S-adenosylmethionine (SAM) as the methyl donor and acetyl-CoA as the acetyl group donor, thereby establishing a direct biochemical link between cellular metabolism and epigenetic regulation (Huo et al., 2021). Beyond methylation and acetylation, several other epigenetic modifications contribute to the dynamic regulation of chromatin structure and gene expression. These include ubiquitination, phosphorylation, SUMOylation, and ADP-ribosylation—mostly targeting histone tails and modifying chromatin accessibility, DNA repair, and transcriptional activity (Li Z. et al., 2023). Additionally, RNA molecules undergo post-transcriptional modifications, such as N6-methyladenosine (m6A), which influence RNA stability, splicing, transport, and translation, with growing interest in their roles in cancer and therapeutic potential (Chen et al., 2025; Bove et al., 2023).

Continuing the description of metabolites that can serve as substrates for epigenetic enzymes, notable examples include succinylation, which depends on succinyl-CoA; lactylation, derived from lactate; and palmitoylation, involving the attachment of palmitic acid (Wu et al., 2024). Additionally, other metabolite-dependent modifications have been identified, such as crotonylation (dependent on crotonyl-CoA), malonylation (dependent on malonyl-CoA), propionylation (dependent on propionyl-CoA), butyrylation (dependent on butyryl-CoA), and glutarylation (dependent on glutaryl-CoA) (Figure 2) (Sabari et al., 2017). Among the range of epigenetic modifications that use metabolites as substrates, some involve the utilization of lipids. Farnesylation and geranylgeranylation are two types of prenylation, a class of lipid modifications involving the covalent attachment of isoprenoid groups. Specifically, a binding of a 15-carbon farnesyl or a 20-carbon geranylgeranyl moiety occurs to cysteine residues near the C-terminus of target proteins. Prenylation is catalyzed by specific prenyltransferases (farnesyltransferase and geranylgeranyltransferase) that utilize farnesyl pyrophosphate (FPP) or geranylgeranyl pyrophosphate (GGPP) as lipid donors, both derived from the mevalonate pathway. The general role of prenylation is pro-tumorigenic. In fact, inhibitors of the enzymes involved in the formation of these epigenetic modifications have demonstrated the ability to induce apoptosis in cancer cells, along with a reduction in both proliferation and invasion (Berndt et al., 2011). Hydroxybutyrylation is a more recently characterized post-translational modification involving the addition of a hydroxybutyryl group to lysine residues on histones and other proteins. This modification depends on the metabolite β-hydroxybutyrate (BHB), a ketone body produced during fasting, ketogenic diets, or metabolic stress. This modification is involved in metastasis formation in cancer (Jiang et al., 2025).

**FIGURE 2:**
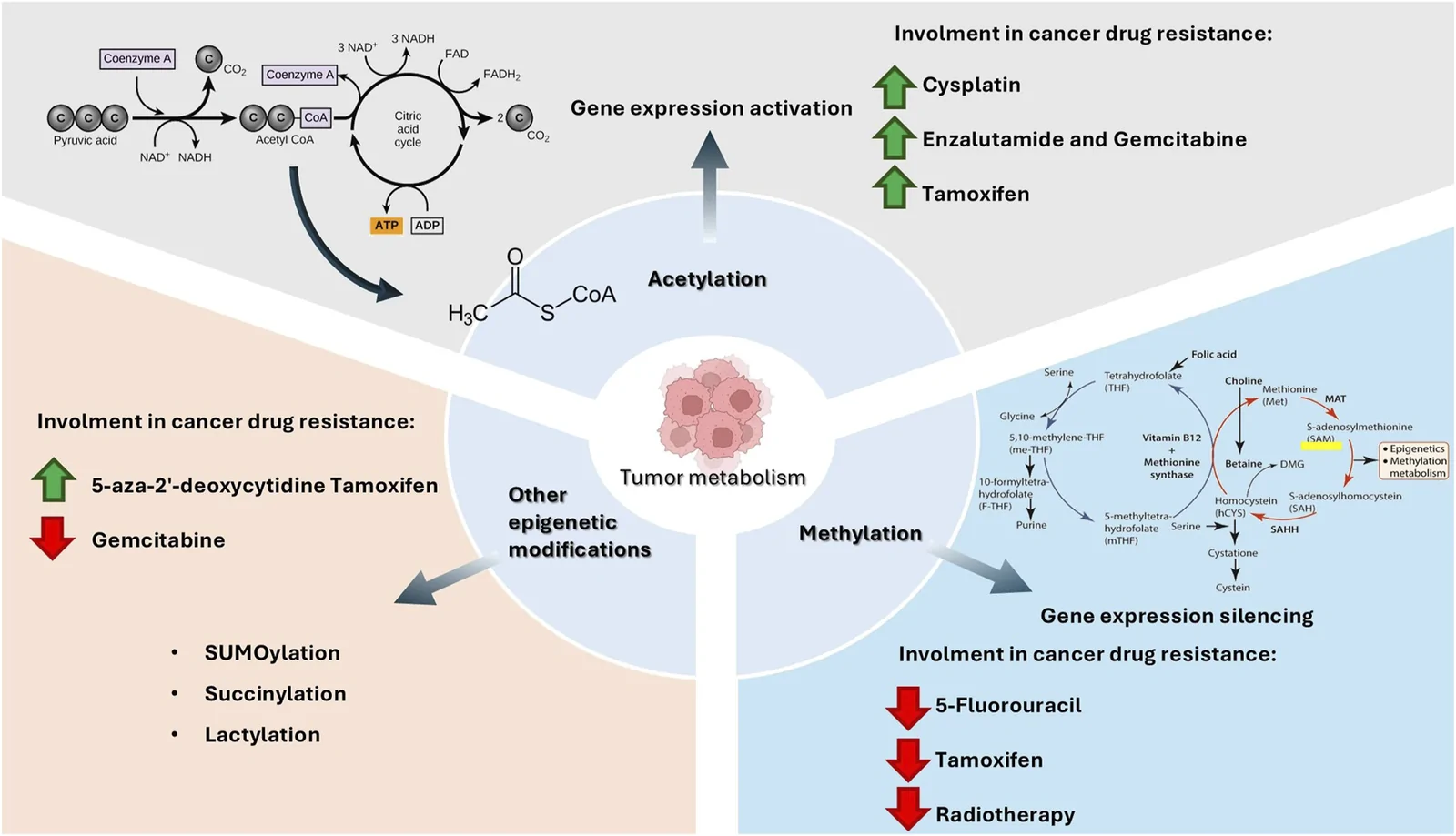
Schematic overview of the role of epigenetic modifications in regulating cancer cell response to therapy. Green arrows indicate cases where an increase in the epigenetic modification is associated with the development of resistance, while the red arrow highlights situations in which a decrease in the modification correlates with therapy resistance.

Epigenetic alterations in cancer generate heterogeneity that promotes the development of resistance, as it increases the likelihood that some cells within the tumor mass will survive therapy or remain in a quiescent state, ultimately leading to disease relapse. Among the epigenetic mechanisms shown to be associated with the development of resistance to chemotherapeutic agents are chromatin remodeling, which can influence the ability of drugs to access DNA, and alterations in microRNAs and long non-coding RNAs, which can in turn regulate genes involved in drug resistance (Mangraviti and Castelli, 2025; Sadida et al., 2023). Dysregulation of enzymes that modulate epigenetic modifications has also frequently been associated with the development of cancer resistance. This is the case for lysine demethylase 5A (KDM5A) and Enhancer of zeste (EZH2), which are upregulated following cisplatin treatment in ovarian and colorectal cancer (Aud et al., 2016). Such evidence has spurred growing interest in epigenetic-targeting agents, including DNMT and HDAC inhibitors, some of which have already received approval for clinical application.

Given the large number of potential epigenetic modifications involved in cancer initiation, progression, and response to therapy, the following sections will summarize recent evidence concerning the most common alterations.

### 3.1 Acetylation in cancer: pro- and anti-tumoral features

Acetyl-CoA is produced by mitochondria and represents a central hub in various metabolic pathways, including the oxidation of glucose, amino acids, and fatty acids. Considering sources of acetylation, glucose is certainly one of the most important. From this perspective, given that glucose represents one of the main energy sources for many tumors, this aspect is particularly relevant in cancer. Lactate also becomes an important source of acetylation in tumors. In the case of lactate, it can be produced endogenously by tumor cells or taken up from the extracellular environment, where it is mainly generated by cells of the tumor microenvironment (Shang et al., 2022). Other important sources of acetyl-CoA include fatty acid β-oxidation and the metabolism of certain amino acids, including branched-chain amino acids (BCAAs), as well as ketone body metabolism. The contribution of acetyl-CoA derived from these different metabolic pathways depends on the cell type, the predominant metabolic program, and the cellular state (Guertin and Wellen, 2023). Beyond the biochemical role, acetyl-CoA represents a critical donor of acetyl groups for post-translational modifications, particularly histone acetylation. As such, fluctuations in acetyl-CoA availability can directly influence chromatin dynamics and gene expression, linking cellular metabolic status to epigenetic regulation and cell fate decisions (Hao et al., 2022). The role of histone acetylation in cancer is not univocal, as acetylation at different residues of various histones can lead to opposing effects on tumor behavior. This variability is also tumor-type dependent. Specifically, regarding histone H3 acetylation, certain modifications are particularly associated with specific cancer types, potentially serving as true biomarkers (Miziak et al., 2024).

Histone acetylation occurs on lysine residues of histone proteins, leading to a reduction in the positive charge of histones and, consequently, a weaker interaction with DNA. This results in a more relaxed chromatin structure, which is more accessible to the transcriptional machinery. As a consequence, genes located in these regions are more easily expressed. In general, therefore, histone acetylation is considered a modification that promotes gene expression (Shvedunova and Akhtar, 2022; Wang and Ma, 2025).

In the context of the response to chemotherapy, histone acetylation has generally been associated with the development of resistance (Figure 2). Gene ontology analysis of differentially expressed genes in cisplatin-resistant tumor cell lines compared to their parental counterparts highlighted the involvement of pathways related to epigenetic modifications. In particular, gene expression alterations observed in cisplatin-resistant HeLa and HepG2 cell lines were evident both at the level of enzymes responsible for epigenetic modifications and in histone acetylation itself. Indeed, histone acetylation appeared to be a crucial factor in the development of resistance to cisplatin (Natu et al., 2024).

Consistently, several histone acetyltransferase (HAT) inhibitors have shown promising effects in targeting cancer stem cells and overcoming drug resistance. For example, pharmacological inhibition of KAT6A in ovarian cancer models has been associated with increased cisplatin sensitivity, suggesting a potential role for HAT inhibitors in combination strategies aimed at overcoming chemoresistance (Liu et al., 2021). In addition, in castration-resistant prostate cancer, in which high expression of histone acetyltransferase 1 (HAT1) can enhance the resistance to enzalutamide and gemcitabine (Wang et al., 2023), inhibitors of histone acetylation have been proposed as therapeutic agents or adjuvant treatments, showing potential in reducing tumor burden. FLIM-FRET screening has identified a panel of 11 potential epigenetic biomarkers for breast cancer therapy. Among these, several were associated with DNA modifications, including methylation and acetylation patterns. Based on these findings, anacardic acid, a natural compound, was subsequently identified as a potential adjuvant to standard Tamoxifen therapy. Its co-administration was found to enhance Tamoxifen efficacy and reduce the likelihood of treatment resistance. Anacardic acid is proposed to exert its effects by inhibiting p300 HAT activity, leading to a reduction in H4K12 and H3K27 acetylation levels and a consequent decrease in occupancy at the transcription start site of estrogen response element (ERE)-regulated genes (Liu et al., 2019). However, for the sake of completeness, it is important to note that HDAC (histone deacetylase) inhibitors may also exert a beneficial effect by enhancing the response to therapy in castration-resistant prostate cancer. In this context, the improved efficacy of chemotherapeutic regimens combined with HDAC inhibitors is largely attributed to their impact on non-histone protein acetylation. Notably, several histone deacetylase (HDAC) inhibitors, including Romidepsin, Vorinostat, and Panobinostat, are currently in clinical development and have progressed to phase II clinical trials (Biersack et al., 2022). A phase I clinical trial is going on to evaluate the safety and maximum tolerated dose of EP31670, a dual inhibitor of BET and CBP/P300, in advanced solid tumors (Wang et al., 2023). This evidence highlights the critical role that intracellular acetylation balance may play in modulating therapeutic outcomes.

Non-histone acetylation occurs on proteins located in the cytosol or other cellular compartments, and plays a key role in regulating the activity and function of these proteins (Wang and Ma, 2025). A prominent example is p53, which can be acetylated by the acetyltransferase p300. This post-translational modification (PTMs) regulates p53 activity; for instance, increased levels of acetylated p53 in hepatocellular carcinoma are associated with a metabolic shift toward oxidative metabolism, resulting in reduced cell proliferation (Di Leo et al., 2019).

Lysine acetyltransferases (KATs) and lysine deacetylases (KDACs) play a central role in the dynamic regulation of protein acetylation by mediating the addition and removal of acetyl groups on both histone and non-histone proteins. KDACs are broadly categorized into two classes based on their cofactor dependency: zinc-dependent histone deacetylases and NAD^+^-dependent sirtuins (Li Z. et al., 2025). From a therapeutic perspective, the inhibition of non-histone acetylation can be achieved through the use of HDAC inhibitors, which, as previously mentioned, are frequently employed in combination with chemotherapeutic agents in cancer treatment. For instance, metformin has been shown to enhance the anti-bladder cancer activity of Panobinostat, likely through AMPK activation and the consequent regulation of the acetylation/deacetylation balance (Okubo et al., 2019). Further supporting the role of non-histone acetylation in therapy resistance, studies have shown that in breast cancer, cisplatin normally induces acetylation of serine-arginine protein kinase 1 (SRPK1). However, in cisplatin-resistant cells, SRPK1 acetylation is diminished, promoting its phosphorylation instead. This post-translational modification alters SRPK1 function, driving the expression of anti-apoptotic splicing variants. This evidence is particularly significant, as it suggests that targeting SRPK1 acetylation may offer a novel therapeutic approach to resensitize resistant breast cancer cells to cisplatin (Wang C. et al., 2020).

### 3.2 Methylation in cancer: pro- and anti-tumoral features

Human cells express more than 100 epigenetic enzymes that are able to modify histones, DNA and transcriptional factors. Most of them require a metabolite for their activity contributing to the emergence of a complex network system. As Wang et al. accurately described, 43 HMTs use SAM as a co-substrate; 2 LSDs that use FAD; and 21 JMJD enzymes that use alpha-ketoglutarate (Kim et al., 2020). Alterations in the DNA methylome have been associated with tumorigenesis across various solid tumors. DNA methylation, which primarily involves the addition of a methyl group to the 5-carbon position of cytosine residues within cytosine-guanine (CpG) dinucleotides, is one of the earliest discovered and most extensively studied epigenetic modifications. In general, DNA methylation leads to gene expression silencing, whereas hypomethylation, particularly in the promoter regions of oncogenes, can promote their overexpression. A similar mechanism has also been linked to genes involved in the response to chemotherapy, highlighting the role of epigenetic regulation in drug sensitivity and resistance (Romero-Garcia et al., 2020; Atlante et al., 2025). Such epigenetic alterations are not always inherited but can be acquired during tumor progression. They may directly affect oncogenes or tumor suppressor genes, or act indirectly through modifications in the promoters of their regulatory elements, such as microRNAs and lncRNAs (Mangraviti and Castelli, 2025; Romero-Garcia et al., 2020). Numerous alterations have been identified at the level of CpG islands, including both hypomethylation and hypermethylation events, and they are associated with drug resistance (Figure 2). In colorectal cancer, DNA methylation profiling has been performed in cells resistant to 5-Fluorouracil (5-FU), a widely used chemotherapeutic agent whose clinical efficacy is often limited by the development of drug resistance. As a result, five genes—CCNE1, CCNDBP1, PON3, CHL1, and DDX43—have been identified and proposed as potential therapeutic targets to overcome 5-FU resistance (Baharudin et al., 2017). Dietary folate intake plays a critical role in regulating methylation metabolism in the colorectal mucosa. Folate deficiency reduces intracellular levels of SAM, leading to global DNA hypomethylation and an increased risk of colorectal cancer. In premalignant or very early stages, limiting one-carbon unit availability can transiently suppress nucleic acid synthesis, impairing the replication capacity of highly proliferative adenomatous cells and exerting a growth-inhibitory effect. However, once tumor transformation has occurred, chronic folate insufficiency compromises thymidylate synthesis efficiency and promotes uracil misincorporation into DNA. This results in increased double-strand breaks and chromosomal instability, accelerating clonal selection and the evolution of more aggressive tumor phenotypes. The folate cycle regulates the intracellular balance between SAM and S-adenosylhomocysteine (SAH), thereby controlling the activity of DNA methyltransferases and histone methyltransferases. While modest increases in SAM levels may help preserve the epigenetic integrity of tumor suppressor gene promoters, excessive methyl-donor availability in a pro-oncogenic signaling environment may stabilize chromatin configurations that favor tumor progression (Sun Y-H. et al., 2025). Thus, the use of methylation modulators has been proposed in colorectal cancer as a strategy to overcome drug resistance. Among these, inhibitors targeting HMTs and histone demethylases (HDMs) are currently under investigation for their potential to suppress colorectal cancer cell proliferation and enhance sensitivity to chemotherapy (Haynes and Manogaran, 2025).

Another example of the close relationship between methylation and drug resistance in cancer is observed in nine esophageal cancer cell lines resistant to Docetaxel, Nedaplatin, Mitomycin, and Cisplatin, hypermethylation of the MCTP1 gene—implicated in signal transduction—has been observed. This hypermethylation leads to a downregulation of gene expression (Kong et al., 2021).

As with other histone modifications, methylation can also contribute to the generation of cancer cell subpopulations. This heterogeneity is not only genetic or epigenetic but also phenotypic, and it can influence how tumor cells respond to anticancer therapies. With regard to methylation, heterogeneous methylation patterns contribute to the formation of tumor cell clones that confer heterogeneity and plasticity to the entire tumor mass, thereby promoting the development of resistant cells as well as the emergence of dormant cells. These cells do not undergo cell death following treatment but instead remain in a non-proliferative state, retaining the potential to be reactivated later under more favorable conditions (Sadida et al., 2024). Numerous studies have demonstrated that promoter hypermethylation of genes such as MLH1, MGMT, and BRCA1 can drive resistance to different anticancer agents (Romero-Garcia et al.; Sadida et al., 2024). For example, in non-small-cell lung cancer, hypermethylation of IGFBP3 is associated with reduced sensitivity to cisplatin, while hypermethylation of the MGMT gene has been shown to influence the response to temozolomide in glioblastoma (Duruisseaux and Esteller, 2018).

The dioxygenase ten-eleven translocation (TET) enzyme promotes DNA demethylation, which is often altered in cancer. These enzymes represent a bridge between the epigenetic modification of methylation and metabolism, as TET enzymes are two-oxoglutarate (also known as α-ketoglutarate)-, oxygen-, and iron-dependent dioxygenases, and they use ascorbic acid as a cofactor (Salmerón-Bárcenas et al., 2024). Loss of TET2 expression has been shown to promote resistance to tamoxifen treatment *in vivo*, by promoting mammary tumor development with deficient ERα expression (Kim et al., 2020). The role of TET enzymes in chemotherapy resistance is compounded by the fact that they can perform functions not strictly associated with methylation. For example, in hepatocellular carcinoma, it has been shown that DNMT3a and TET2 act coordinately to regulate cancer cell fate through both DNA methylation-dependent and -independent mechanisms, supporting drug resistance and poor prognosis. For this reason, they have been proposed as promising therapeutic targets for refractory hepatocellular carcinoma (Cheng et al., 2024).

Histone methylation influences tumor response to radiotherapy primarily through the regulation of DNA damage repair pathways, particularly homologous recombination (HR) and nonhomologous end joining (NHEJ). Specifically, methylation marks on histone residues H3K36 and H3K27me3 are critically involved in these processes (Wen et al., 2023). For instance, trimethylation of H3K36 by the methyltransferase SETD2 is essential for the activation of ATM kinase and the recruitment of 53BP1 to sites of DNA double-strand breaks (DSBs), facilitating effective DNA repair. In contrast, overexpression of the histone demethylase JMJD2A reduces H3K36 methylation, impairs HR repair efficiency, and may thereby sensitize tumor cells to radiation. On the other hand, Metnase, a methyltransferase that also targets H3K36, promotes NHEJ repair, ultimately enhancing radiation resistance (Song et al., 2025).

Given the strong connection between epigenetic modifications and the response of cancer cells to both chemotherapy and radiotherapy, numerous epigenetic modulators have been introduced into cancer treatment as adjuvants to conventional therapies (Table 2). To date, numerous epigenetic modulators have received FDA approval for use in cancer therapy. In addition to FDA-approved agents, a wide range of epigenetic modulators—particularly DNA methyltransferase inhibitors are currently being evaluated in clinical trials across various cancer types. For example, guadecitabine is undergoing phase III trials for acute myeloid leukemia (ClinicalTrials.gov ID NCT02920008), while RX-3117 is being tested in phase I/II studies for metastatic pancreatic and bladder cancers (ClinicalTrials.gov ID NCT03189914). Other DNMTis such as 5-fluoro-2′-deoxycytidine are in phase II trials for head and neck, lung, and breast cancers (ClinicalTrials.gov ID NCT00978250).

**TABLE 2:** Summary table of epigenetic inhibitors or approved drugs and their metabolic targets.

Drug/Compound	Epigenetic target	Metabolic axis involved	Cancer context	References
Azacitidine	DNMT1 inhibition	One-carbon metabolism (SAM/SAH); DNA methylation–dependent transcription	Reactivation of MEG3–p53/PTEN axis	Li et al. (2016)
5-aza-2′-deoxycytidine	DNMT inhibition	DNA methylation programs linked to methyl-donor availability	Combination with SUMO inhibition in hematologic malignancies	Kroonen et al. (2023)
Guadecitabine	DNMT inhibition	DNA methylation	Phase III, AML	Zhang et al. (2022)
RX-3117	DNMT inhibition	DNA methylation	Phase I/II, pancreatic and bladder cancer	Zhang et al. (2022)
5-fluoro-2′-deoxycytidine	DNMT inhibition	DNA methylation	Phase II, solid tumors	Zhang et al. (2022)
Hydralazine	DNA-modifying agent	DNA methylation	Phase III, ovarian cancer	Zhang et al. (2022)
EGCG	DNA-modifying agent	DNA methylation	Phase II, prostate and colon cancer	Zhang et al. (2022)
Genistein	DNA-modifying agent	DNA methylation	Investigational	Zhang et al. (2022)
Curcumin	DNA-modifying agent	DNA methylation	Colorectal and breast cancer	Zhang et al. (2022)
Disulfiram	Repurposed epigenetic-active agent	DNA methylation–related pathways	Repurposed in solid tumors	Zhang et al. (2022)
Resveratrol	Repurposed epigenetic-active agent	DNA methylation–related pathways	Repurposed in solid tumors	Zhang et al. (2022)
Romidepsin, Vorinostat, Panobinostat	HDAC inhibitor	Acetyl-CoA–dependent acetylation; non-histone acetylation	Phase II, castration-resistant prostate cancer	Biersack et al. (2022)
EP31670	BET/CBP–p300 inhibitor	Histone acetylation programs linked to metabolic state	Phase I, advanced solid tumors	Wang et al. (2023)
Anacardic acid	HAT (p300) inhibitor	Reduced H3/H4 acetylation	Adjuvant to tamoxifen (ER^+^ breast cancer)	Liu et al. (2019)
TAK-981	SUMOylation inhibitor	Stress-response and epigenetic regulation	Combination with DNMT inhibitors in MYC-driven hematologic cancers	Kroonen et al. (2023)
Glyburide	Succinyltransferase activity inhibition	Succinyl-CoA–dependent lysine succinylation	Ovarian cancer; reversal of cisplatin resistance	Zhu et al. (2025)

Beyond DNA methyltransferase inhibitors, other compounds with epigenetic or DNA-modifying activity, such as hydralazine, EGCG, genistein, and curcumin, are also under investigation for their potential to enhance the efficacy of conventional therapies. For instance, hydralazine is in phase III trials for ovarian cancer (ClinicalTrials.gov ID NCT00533299), EGCG is in phase II for prostate and colon cancers (ClinicalTrials.gov ID NCT02891538), and curcumin is being evaluated for colorectal and breast cancers (ClinicalTrials.gov ID NCT01042938). Some agents, like disulfiram and resveratrol, are being repurposed for oncology and studied in multiple solid tumors, including breast, lung, and colon cancers (Zhang et al., 2022).

### 3.3 Other epigenetic modifications pro- and anti-tumoral features in regulating drug response

Undoubtedly, acetylation and methylation represent the main epigenetic modifications, both histone and non-histone, on which research has primarily focused in relation to the regulation of cancer therapy response. Beyond these, however, as previously mentioned, other epigenetic modifications may contribute to establishing a strong link between cellular metabolism and tumor aggressiveness (Figure 2).

SUMOylation is a reversible post-translational modification in which small ubiquitin-like modifier proteins (SUMOs) are covalently attached to lysine residues on target proteins through an enzymatic cascade. This modification regulates multiple cellular processes, including protein stability, subcellular localization, protein–protein interactions, transcriptional activity, and the cellular response to stress (Huang et al., 2024). In hematopoietic malignancies, inhibition of SUMOylation has been shown to enhance the activity of DNA hypomethylating agents, thereby increasing drug efficacy and limiting malignant cell expansion. In particular, the combination of the SUMOylation inhibitor TAK-981 and the DNA hypomethylating agent 5-aza-2′-deoxycytidine has been investigated as a promising therapeutic strategy to improve treatment outcomes in MYC-driven hematopoietic cancers (Kroonen et al., 2023).

Lactylation consists of the addition of lactate to proteins, including histones. The lactate used for these modifications is mainly derived from glucose, linking lactylation to glycolytic metabolism. In addition, lactate can originate from the tumor microenvironment, thereby creating a functional link between the epigenetic state of tumor cells and the surrounding microenvironment (Xu et al., 2023). Histone lysine lactylation, which primarily occurs on lysine residues of H2A, H2B, H3, and H4, has frequently been found to be altered in cancer. Elevated global levels of histone lactylation have been observed across most cancer types, where this modification contributes to the stimulation of gene transcription within chromatin. Given the critical role of lactylation in cancer development and resistance to therapy, targeting lactylation pathways represents a promising avenue for new cancer treatments. While most inhibitors targeting lactylation are still at the preclinical research stage, a few have progressed to clinical trials. Notably, compounds such as AZD3965, an inhibitor of MCT1, and 2-deoxy-D-glucose (2-DG), a glycolysis inhibitor, have undergone evaluation for their safety profiles, pharmacokinetics, and maximum tolerated doses in clinical settings (Sun Y. et al., 2025).

Lysine succinylation is a recently identified post-translational modification characterized by the covalent addition of succinyl groups to lysine residues on target proteins, thereby modulating their structure, activity, and function. Succinyl-coenzyme A (succinyl-CoA) represents the primary donor of succinyl groups, and its intracellular levels are tightly regulated by multiple metabolic pathways. These include the TCA cycle, BCAA catabolism, and the β-oxidation of fatty acids. Most cellular succinylation reactions are enzymatically regulated by the coordinated activity of succinyltransferases and desuccinylases. Through the balanced action of these opposing enzymes, both histone and non-histone proteins undergo dynamic succinylation and desuccinylation (Zheng et al., 2020). In ovarian cancer, MFF succinylation promotes the maintenance of stemness and contributes to cisplatin resistance. Glyburide was used to inhibit lysine succinyltransferase activity and block MFF succinylation (Zhu et al., 2025). Histone succinylation has also been linked to chemotherapy resistance. For example, in prostate cancer, increased levels of H3K122 succinylation have been observed. Conversely, in pancreatic cancer, elevated H3K122succ levels have been associated with increased sensitivity of cancer cells to gemcitabine (Gao and Yu, 2025).

These modifications belong to a recently classified novel class of histone “non-enzymatic covalent modifications” (NECMs), which lies at the intersection between epigenetics and metabolic fitness and plays a role in cancer, particularly in the response to therapy.

In this context, the link with metabolism is direct, as these modifications are not enzymatically driven but instead depend solely on the concentration of the metabolite. The metabolites responsible for these histone modifications are generally produced in the cytoplasm through different metabolic pathways primarily aimed at ATP generation. When the concentration of specific intermediates increases, they can freely diffuse into the nucleus, where they covalently bind to histone proteins (Zheng et al., 2020) (Figure 3).

**FIGURE 3:**
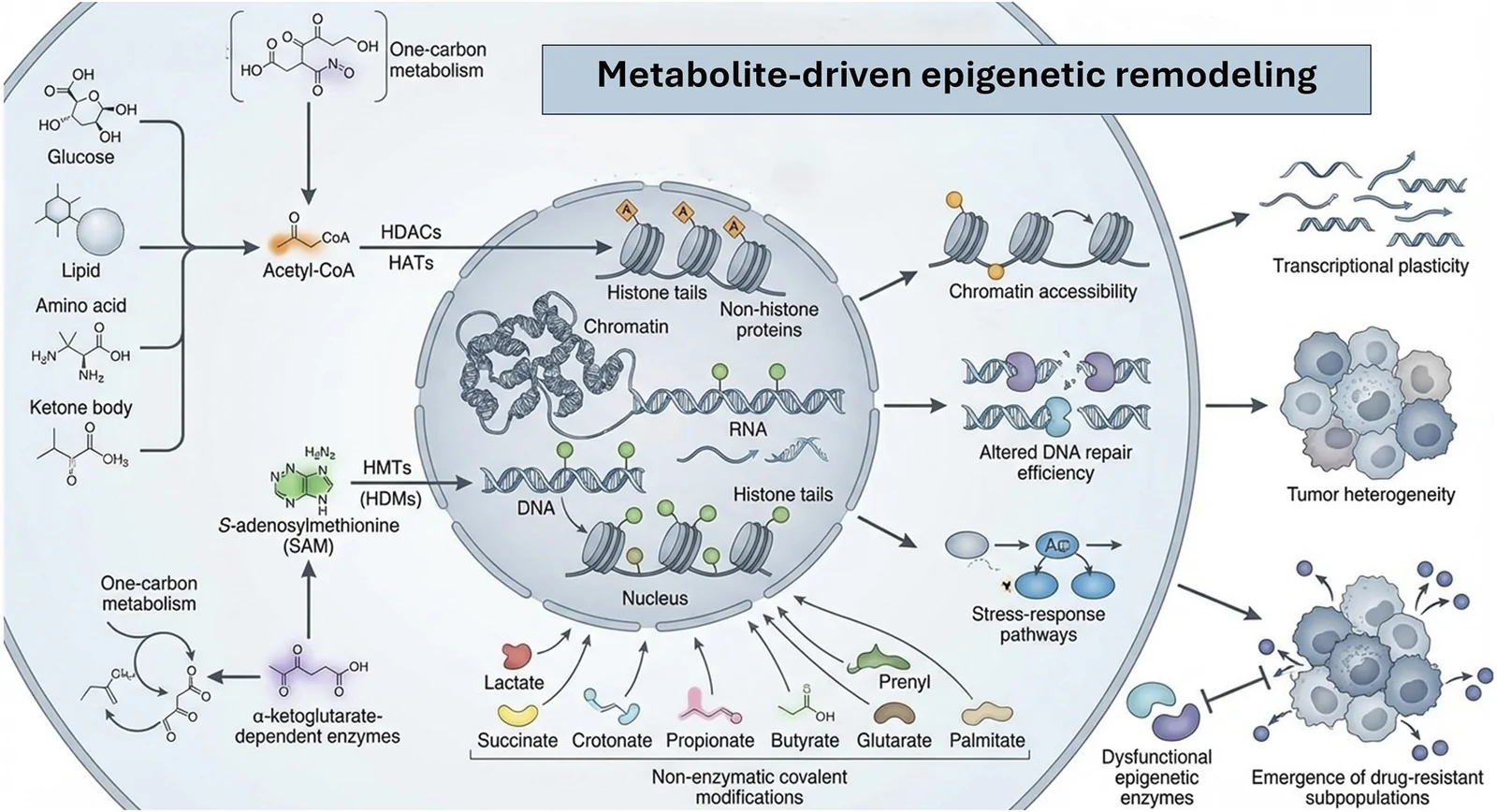
Schematic representation of the bidirectional interplay between cellular metabolism and epigenetic regulation in cancer. Metabolic pathways, including glucose, lipid, amino acid, and ketone body metabolism, generate key metabolites such as acetyl-CoA, S-adenosylmethionine (SAM), α-ketoglutarate, lactate, succinate, and lipid-derived intermediates. These metabolites act as substrates or cofactors for epigenetic enzymes, regulating DNA, RNA, histone, and non-histone protein modifications, including acetylation, methylation, and other metabolite-dependent post-translational modifications. Metabolite availability directly influences chromatin accessibility, transcriptional activity, DNA repair efficiency, and stress-response pathways. The resulting epigenetic reprogramming promotes intratumoral heterogeneity and supports the emergence of drug-tolerant and therapy-resistant cancer cell subpopulations, highlighting the epigenetic–metabolic axis as a key determinant of tumor adaptation and therapeutic response.

Because these modifications, as mentioned above, are associated with the aggressiveness of many tumors, several therapeutic strategies have been developed and tested to regulate NECMs. These approaches are mainly based on targeting erasers, which are responsible for removing the modifications, and readers, which recognize and interpret them (Scumaci and Zheng, 2023; Maksimovic and David, 2021).

## 4 Bioinformatics and emerging frontiers in epigenetics-based drug discovery

Epigenetics is rapidly advancing as a frontier in drug discovery. Multi-omics technologies now allow the mapping of intricate regulatory networks that sustain disease, and progress in the field increasingly depends on the integration of diverse data layers to identify drug targets, predict therapeutic responses, and anticipate resistance.

### 4.1 Major multi-omics resources for epigenetic and metabolic studies

The Cancer Genome Atlas (TCGA) has transformed cancer research by providing integrated genomic, transcriptomic, epigenomic, and proteomic profiles from over 20,000 tumors and matched normal tissues across 33 cancer types (Cancer Genome Atlas Research Network et al., 2013). The completion of the Pan-Cancer Atlas expanded these resources by uncovering molecular commonalities and lineage-specific differences across tumor types (Hoadley et al., 2018). Analyses of TCGA datasets have revealed epigenetic vulnerabilities and metabolic dependencies with therapeutic potential (Lehmann et al., 2021; Rosario et al., 2018), demonstrated how DNA methylation and histone modifications open new treatment avenues (Gnad et al., 2015; Cheng et al., 2023), and guided the development of targeted epigenetic therapies (Fan et al., 2019).

Complementing TCGA, the Encyclopedia of DNA Elements (ENCODE) provides the regulatory context with nearly 6,000 datasets spanning chromatin accessibility, transcription factor binding, histone modifications, DNA methylation, and regulatory element activity across diverse cellular states (ENCODE Project Consortium, 2012; ENCODE Project Consortium et al., 2020; Luo et al., 2020; Subramanian et al., 2020). These data are critical for designing combination therapies that leverage epigenetic drugs to sensitize tumors to conventional treatments or bypass resistance (Klemm et al., 2019).

The Gene Expression Omnibus (GEO) serves as a central repository for epigenetic drug–relevant datasets, housing thousands of studies on drug responses, biomarkers, and resistance mechanisms (Barrett et al., 2013). With over five million samples, GEO now includes curated pharmacoepigenomics signatures that enable target validation, biomarker discovery, and patient stratification (Wang et al., 2016; Wang et al., 2019).

Extending from these descriptive resources, the Cancer Dependency Map (DepMap) provides functional insights by integrating CRISPR-Cas9 screening with multi-omics profiling (Tsherniak et al., 2017). DepMap has uncovered synthetic lethal interactions involving epigenetic regulators, revealing opportunities for precision therapies (Behan et al., 2019). Recent analyses highlight lineage-specific vulnerabilities and show how epigenetic states shape therapeutic susceptibility and resistance (Ohnmacht et al., 2023).

Taken together, these resources provide raw data landscapes for epigenetics-based drug discovery. To extract actionable insights, however, computational integration tools are essential.

### 4.2 Computational integration tools for drug discovery

Weighted Gene Co-expression Network Analysis (WGCNA) identifies drug targets by constructing co-expression modules that capture coordinated epigenetic and metabolic responses to therapy (Langfelder and Horvath, 2008; Zhang et al., 2005). Applied to TCGA, WGCNA has revealed modules linked to drug sensitivity, informing network-based biomarkers for patient stratification (Pei et al., 2017).

The Algorithm for the Reconstruction of Accurate Cellular Networks (ARACNe) reconstructs transcriptional regulatory networks from multi-omics data (Margolin et al., 2006). By identifying master regulators affected by epigenetic drugs, ARACNe reveals targets, feedback loops, and resistance mechanisms, guiding combination strategies.

iClusterPlus enables integrative subtyping by combining genomic, epigenomic, and transcriptomic data (Shen et al., 2009). It has been applied to stratify patients, predict resistance, and uncover therapeutic vulnerabilities, particularly in hematologic malignancies treated with agents such as 5-azacytidine and HDAC inhibitors (Lachmann et al., 2016; Califano and Alvarez, 2017).

When applied in concert, these tools make it possible to move from descriptive datasets to functional insights, supporting the development of precision therapies (Table 3).

**TABLE 3:** Summary table of the potential usefulness of bioinformatic tools in the context of drug discovery and drug resistance.

Category	Tool	Primary function	Application to drug discovery	Key references
Cancer multi-omics resources	TCGA/pan-cancer atlas	Genomic, transcriptomic, epigenomic, proteomic profiling across 33 tumor types	Identifies epigenetic vulnerabilities, metabolic dependencies, biomarkers for therapy response and resistance	Cancer Genome Atlas Research Network et al. (2013), Hoadley et al. (2018), Lehmann et al. (2021), Rosario et al. (2018), Gnad et al. (2015), Fan et al. (2019)
Regulatory epigenomic mapping	ENCODE	Mapping chromatin accessibility, TF binding, histone marks, regulatory elements	Builds epigenetic regulatory maps that guide combination epigenetic therapies and resistance prediction	ENCODE Project Consortium (2012), ENCODE Project Consortium et al. (2020), Luo et al. (2020), Subramanian et al. (2020)
Drug-response transcriptomics repository	GEO	Public repository of transcriptomic, epigenomic, and drug-response datasets	Provides pharmacoepigenomic signatures, biomarker discovery, target validation	Barrett et al. (2013), Wang et al. (2016), Wang et al. (2019)
Functional genomics	DepMap	CRISPR knockout + multi-omics integration	Identifies synthetic lethality involving epigenetic regulators; lineage-specific metabolic dependencies	Barrett et al. (2013), Behan et al. (2019), Ohnmacht et al. (2023)
Network biology	Weighted gene co-expression Analysis (WGCNA)	Builds co-expression modules	Identifies ncRNA–metabolic modules linked to drug sensitivity; stratifies patients	Langfelder and Horvath (2008), Zhang et al. (2005), Pei et al. (2017)
Regulatory network inference	ARACNe	Reconstructs transcriptional regulatory networks	Identifies master regulators affected by epigenetic drugs; reveals feedback loops and resistance nodes	Margolin et al. (2006), Lachmann et al. (2016), Califano and Alvarez (2017)
Integrative clustering	iClusterPlus	Integrates genomics, epigenomics, transcriptomics	Identifies epigenetic–metabolic subtypes; predicts resistance to DNMT inhibitors and HDAC inhibitors	Shen et al. (2009), Mo et al. (2013), Meng et al. (2016)
ncRNA–metabolite association mapping	xMWAS/DIABLO	Multi-omics correlation of ncRNAs, mRNAs, metabolites	Links lncRNAs to metabolic enzyme regulation; identifies ncRNA metabolic hubs	Singh et al. (2019)
ncRNA–metabolite AI modeling	mmvec	Neural embedding of microbial or metabolite co-occurrence	Captures ncRNA–metabolite interactions predicting metabolic rewiring	Morton et al. (2019)
AI-based epigenetic–metabolic prediction	MER-Net	Deep learning on epigenomic + metabolic networks	Predicts metabolic states from chromatin features	Ge et al. (2022)
Genome-scale network integration	Integrated metabolic + regulatory networks	Predict essential metabolic genes, synthetic lethal pairs	Identifies targetable metabolic vulnerabilities linked to chromatin states	Barrena et al. (2023)
Drug repurposing platforms	DepMap drug response, PRISM, Connectivity map/LINCS	Drug sensitivity screens + perturbation signatures	Predict compounds reversing epigenetic–metabolic dysregulation; nominate drug combinations	Ghandi et al. (2019), Meyers et al. (2017), Subramanian et al. (2017), Pujalte-Martin et al. (2024)
Graph-based AI for drug discovery	Heterogeneous graph learning approaches	Integrate genes, drugs, metabolites, ncRNAs, diseases	Nominates repurposable drugs targeting epigenetic–metabolic vulnerabilities	Zhao et al. (2022), You et al. (2022)
AI-driven multi-omics for epigenetic targets	Deep neural classifiers on multi-omics	Predict metabolic dependencies from chromatin	Identifies therapeutic candidates targeting histone-modifier mutations	Salvati et al. (2025)

### 4.3 Network biology and AI/ML approaches

Systems biology now seeks to integrate molecular, cellular, and tissue processes, particularly the coupling of epigenetic regulation (DNA methylation, histone modifications, chromatin architecture) with metabolic states (enzyme expression, flux, metabolite pools). Network biology provides a natural scaffold, representing genes, ncRNAs, proteins, chromatin elements, and metabolites as nodes, and their regulatory or biochemical relationships as edges. Multi-omics datasets enable multiplex networks that explicitly encode cross-talk among molecular layers.

Artificial intelligence (AI) offers powerful tools to decode these networks. By learning compact representations of complex, nonlinear multi-omics data, AI can capture cross-layer regulatory loops and context-specific rewiring. These embeddings support prediction, classification, and mechanistic inference, expanding the toolkit for drug discovery.

These concepts become especially powerful when applied to specific biological questions, such as ncRNA regulation of metabolism or the prediction of metabolic reprogramming from chromatin states.

### 4.4 Identifying regulatory ncRNA–Metabolite interactions

Network analyses are pivotal for uncovering ncRNA regulation of metabolism. Co-expression tools like WGCNA identify ncRNA–enzyme modules, while ARACNe infers direct ncRNA–gene regulatory interactions. Methods such as xMWAS and DIABLO extend these insights by correlating ncRNAs, mRNAs, and metabolites, highlighting candidate regulatory axes (Singh et al., 2019). Neural models such as mmvec further capture ncRNA–metabolite co-occurrence patterns (Morton et al., 2019). Together, these approaches reveal ncRNAs acting as metabolic “hubs,” sequestering miRNAs or modulating enzyme expression (Li M. et al., 2025). Similar frameworks can also be applied to understand how chromatin dynamics themselves encode and predict metabolic states.

### 4.5 Predicting metabolic shifts from chromatin dynamics

Chromatin features (DNA methylation, histone modifications, accessibility) both reflect and shape metabolism, enabling multi-omics inference of metabolic phenotypes (Ge et al., 2022). MER-Net exemplifies this integration, linking epigenomic and metabolic networks with deep learning (Wang et al., 2021). Tools like iClusterPlus identify latent epigenetic–metabolic clusters (Sathyanarayanan et al., 2019), while genome-scale metabolic and regulatory network integration predicts essential metabolic genes and synthetic lethal pairs (Barrena et al., 2023). AI-driven classifiers can now predict metabolic reprogramming (e.g., glycolysis vs. oxidative metabolism) directly from chromatin states. These predictive frameworks naturally feed into drug discovery, where the goal is to identify compounds that exploit epigenetic–metabolic vulnerabilities.

### 4.6 Drug repurposing and in silico screening of epigenetic–metabolic targets

Large-scale resources such as DepMap (Ghandi et al., 2019; Meyers et al., 2017), PRISM drug responses, and Connectivity Map/LINCS (Subramanian et al., 2017) form the backbone for network- and AI-driven drug repurposing. Graph-based learning applied to heterogeneous networks of genes, ncRNAs, metabolites, proteins, drugs, and diseases has successfully nominated novel drug candidates (Zhao et al., 2022). Such approaches suggest, for example, that metabolic enzyme inhibitors may be effective in tumors with specific epigenetic profiles (You et al., 2022).

Functional genomics further reveals actionable vulnerabilities: DepMap identifies synthetic lethalities between epigenetic regulators and metabolic pathways (Barrena et al., 2023), while CMap/LINCS nominates compounds that mimic or reverse epigenetic–metabolic dysregulation (Pujalte-Martin et al., 2024). AI applied to multi-omics cohorts connects mutations in histone modifiers to metabolic dependencies and suggests existing inhibitors as therapeutic options (Salvati et al., 2025). These computational screens generate prioritized compound lists for experimental testing, aiming to align epigenetic–metabolic therapies with responsive patient subgroups.

## 5 Conclusion and perspective

The convergence between epigenetic regulation and metabolic remodeling defines one of the most consequential axes of tumor adaptability. Across microRNAs, long non coding RNAs, circular RNAs and chromatin modifying systems, the evidence reviewed here demonstrates that noncoding and epigenetic regulators shape metabolic flux, redox balance, mitochondrial function and nutrient utilization in ways that directly influence drug response. These interactions create self-reinforcing loops that stabilize drug tolerant states, adjust apoptotic thresholds and enable metabolic plasticity during therapeutic pressure. At the same time, several non-coding RNAs including MEG3, IDH1 AS1, LINC PINT and GAS5 illustrate the potential of restoring epigenetic or metabolic restraint to resensitize tumors, emphasizing the dual nature of the epigenetic metabolic interface.

Therapeutically, interventions that modulate DNA methylation, histone acetylation and mutant metabolic enzymes have already shown that targeting this interface can expose hidden vulnerabilities. Yet the adaptability of malignant cells, combined with the strong tissue specificity of many non-coding RNAs, highlights the limitations of strategies that focus on a single molecular node.

Moreover, tumor heterogeneity represents a major barrier to durable therapeutic responses and is increasingly recognized as a consequence not only of genetic diversification but also of spatially resolved metabolic and epigenetic plasticity. Within solid tumors, uneven vascularization and fluctuating nutrient availability generate metabolic gradients characterized by regional differences in oxygen tension, glucose supply, lactate accumulation, redox state, and pH (Mangraviti and Castelli, 2025). These gradients create distinct metabolic microenvironments that act as selective pressures, shaping epigenetic states and transcriptional programs in a context-dependent manner. Accumulating evidence indicates that such metabolic heterogeneity can translate into epigenetic niches within the tumor mass (Mancini et al., 2021). Indeed, this creates metabolite-driven differences in chromatin accessibility, histone post-translational modifications, and non-coding RNA expression that contribute to the emergence of transcriptionally and phenotypically distinct cellular states. Importantly, non-coding RNAs appear to play a central role in stabilizing these niche-specific programs. As discussed throughout this review, lncRNAs and other ncRNA species integrate metabolic cues with chromatin remodeling and metabolic pathway regulation. Within metabolically stratified tumor regions, ncRNA-mediated scaffolding and post-transcriptional control may reinforce localized epigenetic states, thereby sustaining adaptive phenotypes under therapeutic pressure.

Multi omics approaches that integrate transcriptional, chromatin, metabolic and non-coding RNA layers are beginning to map these dependencies with increasing precision, revealing tumor specific epigenetic metabolic signatures and nominating rational therapeutic combinations that may overcome compensatory metabolic remodeling.

Translating these insights into clinical benefit will require patient stratified strategies, biomarkers capable of capturing epigenetic metabolic states and clinical trials designed to test synthetic lethality or cooperative pathway inhibition with precision. Advances in systems biology, artificial intelligence-based modeling, nanoparticle and viral delivery platforms and RNA targeting technologies including small interfering RNAs, antisense oligonucleotides, CRISPR based repression and plasmid driven approaches are positioned to accelerate this transition.

Taken together, the interplay between epigenetic remodeling and metabolic adaptation represents both a major barrier and a powerful therapeutic opportunity in the context of drug resistance. By embracing the complexity of this regulatory axis and leveraging emerging technologies that can dissect and modulate it, it may become possible to convert tumor plasticity from an obstacle into a therapeutic entry point. As mechanistic understanding continues to expand, the epigenetic metabolic circuitry that once drove treatment failure may become a framework for more durable and precisely tailored cancer therapies.
